# Disulfiram Abrogates Morphine Tolerance—A Possible Role of µ-Opioid Receptor-Related G-Protein Activation in the Striatum

**DOI:** 10.3390/ijms22084057

**Published:** 2021-04-14

**Authors:** Anna de Corde-Skurska, Pawel Krzascik, Anna Lesniak, Mariusz Sacharczuk, Lukasz Nagraba, Magdalena Bujalska-Zadrozny

**Affiliations:** 1 Department of Pharmacodynamics, Centre for Preclinical Research and Technology, Medical University of Warsaw, Banacha 1b Str., 02-097 Warsaw, Poland; adecorde@wum.edu.pl (A.d.C.-S.); anna.lesniak@wum.edu.pl (A.L.); 2 Department of Experimental and Clinical Pharmacology, Centre for Preclinical Research and Technology, Medical University of Warsaw, Banacha 1b Str., 02-097 Warsaw, Poland; pkrzascik@wum.edu.pl; 3 Department of Experimental Genomics, Institute of Genetics and Animal Biotechnology in Jastrzebiec, Polish Academy of Sciences, Postepu 36A Str., 05-552 Magdalenka, Poland; m.sacharczuk@ighz.pl; 4 Department of Orthopaedics and Rehabilitation, Medical University of Warsaw, Bursztynowa 2 Str., 04-749 Warsaw, Poland; lukasz.nagraba@wum.edu.pl

**Keywords:** disulfiram, analgesic tolerance, μ-opioid receptor, G-proteins, striatum

## Abstract

One of the key strategies for effective pain management involves delaying analgesic tolerance. Early clinical reports indicate an extraordinary effectiveness of off-label disulfiram—an agent designed for alcohol use disorder—in potentiating opioid analgesia and abrogation of tolerance. Our study aimed to determine whether sustained µ-opioid signaling upon disulfiram exposure contributes to these phenomena. Wistar rats were exposed to acute and chronic disulfiram and morphine cotreatment. Nociceptive thresholds were assessed with the mechanical Randal-Selitto and thermal tail-flick tests. µ-opioid receptor activation in brain structures important for pain processing was carried out with the [^35^S]GTPγS assay. The results suggest that disulfiram (12.5–50 mg/kg i.g.) augmented morphine antinociception and diminished morphine (25 mg/kg, i.g.) tolerance in a supraspinal, opioid-dependent manner. Disulfiram (25 mg/kg, i.g.) induced a transient enhancement of µ-opioid receptor activation in the periaqueductal gray matter (PAG), rostral ventromedial medulla (RVM), hypothalamus, prefrontal cortex and the dorsal striatum at day 1 of morphine treatment. Disulfiram rescued µ-opioid receptor signaling in the nucleus accumbens and caudate-putamen 14 days following morphine and disulfiram cotreatment. The results of this study suggest that striatal µ-opioid receptors may contribute to the abolition of morphine tolerance following concomitant treatment with disulfiram.

## 1. Introduction

Management of refractory pain often involves long-lasting exposure of patients to opioids, which have a long history of mainstay medicine for effective relief of moderate to severe pain [1,2,3]. A plethora of different factors influence the extent of opioid tolerance development. Among molecular mechanisms, downregulation of cell surface receptors, effector uncoupling and desensitization, impaired receptor trafficking, intrinsic drug efficacy and intensity of nociceptive input are the most frequently mentioned. Additionally, the influence of other factors such as dosing regimen, route of administration, drug half-life and plasma concentration were described as important contributing factors (see [4]). Psychological factors have also been shown to play a part in the phenomenon of analgesic tolerance. Unfortunately, a significant percentage of opioid-treated chronic pain patients do not adhere to the prescribed pain management regimen mostly because of their incapability to cope with pain-evoked physical and emotional distress. This misuse of opioids may precipitate the development of hyperalgesia or tolerance as recent studies have shown [5]. Strategies to overcome opioid tolerance include careful drug titration, dose escalation, opioid rotation or use of adjunctive drugs to reduce opioid consumption [6]. A broad selection of adjuncts are available for opioid therapy, including antidepressants [7], anticonvulsants [8], nonsteroidal anti-inflammatory drugs [9], calcium channel blockers [10], NMDA receptor antagonists [11], cholinesterase inhibitors and allosteric modulators of the glutamate receptor 7 (mGlu7) [12,13]. As adjuncts to opioid therapy are used with the key intention of potentiating analgesia and only indirectly affect the development of tolerance only by reducing opioid consumption, their use in a chronic opioid use setting may prove insufficient in the long run [14,15] Additionally, patients often display variability in response to available treatment schemes, thus, introducing novel adjuncts to opioid therapy is one step towards tailoring pain management to the patients’ individual needs.

For decades, disulfiram (DSF) has been used for the pharmacological support of abstinence in patients with chronic alcohol use disorder [16]. By inhibiting acetaldehyde dehydrogenase, it impairs alcohol metabolism and causes buildup of toxic acetaldehyde which causes unpleasant hangover-like effects immediately following ingestion of even small amounts of alcohol [17]. Interestingly, off-label DSF was shown to produce clinically relevant enhancement of stimulation-induced analgesia in refractory pain patients that was insensitive to the development of tolerance for a period of 10 months [18]. The possible mechanistic underpinnings of this phenomenon have not yet been clarified, but several hypotheses were put forward. One hypothetical mechanism proposed by authors in early studies involves enhancement of dopamine release by inhibition of β-hydroxylase [19,20,21]. Of note, dopamine, along with the opioid system, was evidenced as an important player in nociceptive processing as the two neurotransmitter systems topographically overlap and interact in many ways [22,23,24]. Additionally, DSF is converted to many active metabolites that together affect the release of other neurotransmitters such as γ-aminobutyric acid (GABA) and glutamate, which are important mediators of opioid analgesia, withdrawal and tolerance [25,26,27,28]. Another more recent hypothesis assumes that opioid tolerance arises from biased signaling featuring enhanced µ-opioid receptor uncoupling from the G_i/0_ protein and its coupling to the G_s_ protein [29].

Hence, in the present study, we aimed to investigate the possible influence of chronic DSF treatment in morphine (MRF)-tolerant rats on µ-opioid receptor associated G_i/0_ signaling in brain structures important for pain processing.

## 2. Results

### 2.1. The Effect of Disulfiram on Mechanical and Thermal Thresholds

**Figure 1:**
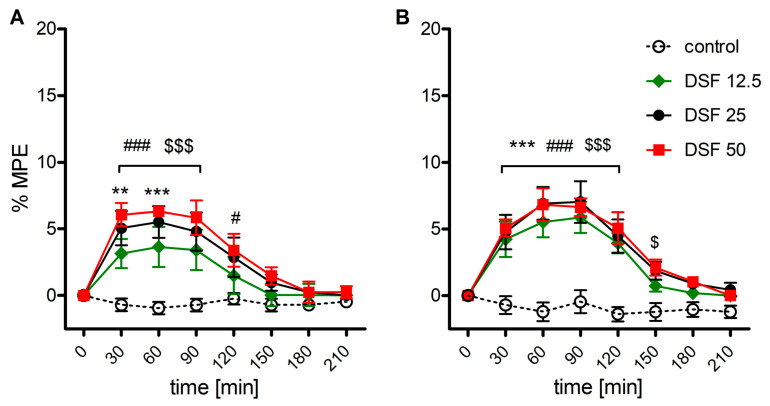
Time-course of the antinociceptive effect of disulfiram (DSF, 12.5-50 mg/kg, i.g) in the Randal-Selitto (**A**) and tail-flick (**B**) tests (*n* = 6). Control animals received 0.1% methylcellulose. Results were expressed as means ± SEM and analyzed with two-way ANOVA followed by Bonferroni’s post hoc test. Statistical differences between control values and DSF treatment groups were indicated as follows: ** *p* < 0.01; *** *p* < 0.001 (DSF 12.5 vs. control); # *p* < 0.05; ### *p* < 0.001 (DSF 25 vs. control); $ *p* < 0.05; $$$ *p* < 0.001 (DSF 50 vs. control).

As shown by two-way ANOVA, a single delivery of disulfiram weakly but significantly increased nociceptive thresholds in response to both mechanical (F_3,160_ = 28.9; *p* < 0.001, Figure 1A) and thermal (F_3,160_ = 50.9; *p* < 0.001, Figure 1B) stimuli. The effect amounting to less than 10% maximum possible effect (%MPE) was dose-independent, surfaced 30 min postdelivery and remained stable for 90 min (mechanical stimulus) or 120 min (thermal stimulus).

### 2.2. The Effect of Disulfiram on Morphine-Induced Antinociception

**Figure 2:**
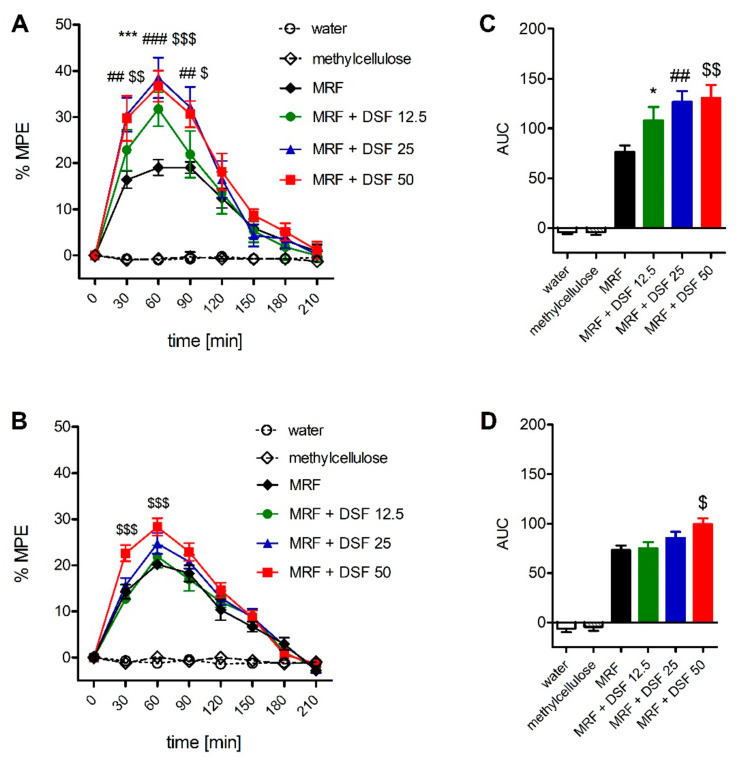
Time-course curves (**A**,**B**) and area under the curve (AUC) (**C**,**D**) of the antinociceptive effect of disulfiram (DSF, 12.5-50 mg/kg, i.g) and morphine (MRF, 25 mg/kg, i.g) cotreatments measured in the Randal–Selitto (**A**) and tail-flick (**B**) tests (*n* = 6). Control animals received either water for injection (MRF group control) or 0.1% methylcellulose (MRF + DSF group control). Results were expressed as means ± SEM and analyzed with two-way ANOVA followed by Bonferroni’s post hoc test (time-course curve data) or one-way ANOVA followed by Dunnett’s post hoc test (AUC data). Statistical differences between MRF and MRF + DSF treatment groups were indicated as follows: * *p <* 0.05; *** *p* < 0.001 (MRF + DSF 12.5 vs. MRF); ## *p* < 0.01; ### *p* < 0.001 (MRF + DSF 25 vs. MRF); $ *p* < 0.05; $$ *p* < 0.01; $$$ *p* < 0.001 (MRF + DSF 50 vs. MRF).

When disulfiram was coadministered with morphine, augmentation of the antinociceptive effect was observed in response to mechanical (F_4,200_ = 57.9; *p* < 0.001, Figure 2A) and thermal (F_4,200_ = 119.7; *p* < 0.001, Figure 2B) stimuli. However, the effect was significant (30–90 min postadministration) for all DSF doses, only when mechanical stimulation was used. When the area under curve (AUC) values were calculated, each dose of DSF potentiated MRF-induced antinociception by 1.4-, 1.6- and 1.7-fold, respectively (Figure 2D). However, when animals were exposed to thermal noxious stimulation, only the highest DSF dose elicited a response at 30–60 min postadministration with AUC values only 1.4-fold higher than in the MRF alone group (Figure 2C). In the Randal–Selitto test, DSF augmented MRF antinociception in a synergistic manner at any dose tested (12.5–50 mg/kg) with CI values of 0.69, 0.61 and 0.66, respectively (Table 1). In the tail-flick test, very weak synergy was observed for the highest dose of DSF (CI = 0.9). When MRF was administered together with 12.5 and 25 mg/kg DSF, no synergistic or additive effect was observed (CI = 1.12 and 1.04, respectively).

### 2.3. Involvement of the Opioid System in the Effect of Disulfiram (DSF)

**Figure 3:**
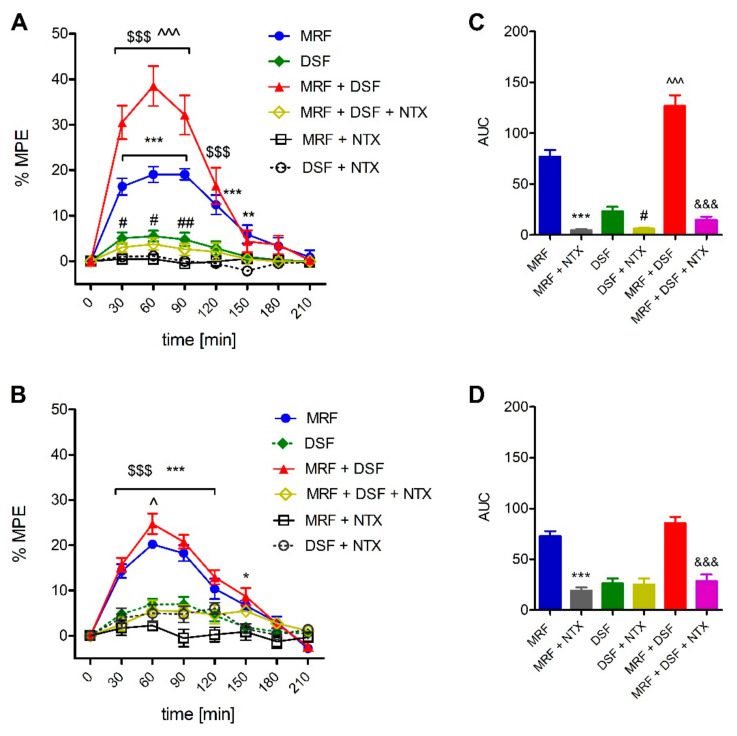
Time-course curves (**A**,**B**) and area under the curve (AUC) (**C**,**D**) of the effect of naltrexone (NTX, 25 mg/kg, i.g) on the antinociceptive effect of morphine (MRF), disulfiram (DSF) and morphine and disulfiram cotreatment (MRF + DSF) in the Randal-Selitto (**A**) and tail-flick (**B**) tests (*n* = 6). Control animals received either water for injection (MRF and MRF + NTX group control) or 0.1% methylcellulose (control for groups treated and cotreated with DSF). Results were expressed as control-subtracted means ± SEM and analyzed with two-way ANOVA followed by Bonferroni’s post hoc test (time-course curve data) or one-way ANOVA followed by Dunnett’s post hoc test (AUC data). Statistical differences between treatment groups and respective controls were indicated as follows: * *p* < 0.05; ** *p* < 0.01; *** *p* < 0.001 (MRF vs. MRF + NTX); # *p* < 0.05; ## *p* < 0.01 (DSF vs. DSF + NTX); $$$ *p* < 0.001 (MRF + DSF + NTX vs. DSF + MRF); *^ p <* 0.05; ^^^ *p* < 0.001 (MRF + DSF vs. MRF); &&& *p* < 0.001.

As shown in Figure 3, naltrexone (NTX) antagonized the antinociceptive effect of MRF and DSF given alone as well as in the MRF + DSF groups when assessed in the Randal–Selitto test (F_5,240_ = 132.6; *p* < 0.001) (Figure 3A,C). However, in the tail-flick test, NTX-induced reversal was seen in MRF and MRF + DSF rats, but not in rats treated with DSF alone (F_5,240_ = 66.8; *p* < 0.001) (Figure 3B,D).

### 2.4. The Effect of Disulfiram on Morphine Tolerance

**Figure 4:**
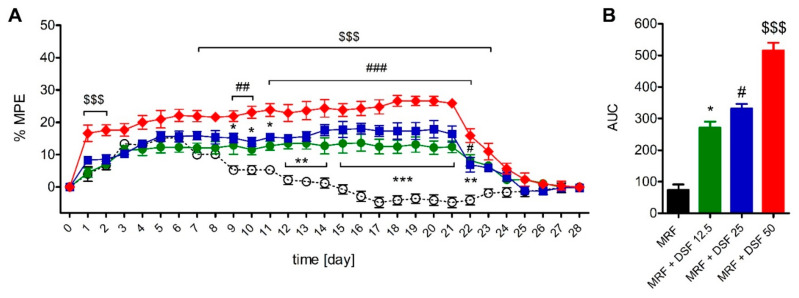
The time-course (**A**) and area under the curve (AUC) (**B**) of chronic disulfiram (DSF, 12.5-50 mg/kg, i.g) on morphine-induced (MRF, 25 mg/kg, i.g) tolerance in the Randal-Selitto test (*n* = 6). ○ MRF; ● MRF + DSF 12.5, ■ MRF + DSF 25, ♦ MRF + DSF 50. Control animals received either water for injection (MRF group control) or 0.1% methylcellulose (MRF + DSF group control). Baseline thresholds were measured at time 0. Drugs were delivered consecutively from day 1 until day 21. Drugs were discontinued on day 22 and nociceptive thresholds were measured until day 28. Results from the time-course curves were expressed as control-subtracted means ± SEM and analyzed with two-way ANOVA followed by Bonferroni’s post hoc test. Area under the curve (AUC) data were analyzed with the Kruskal-Wallis test. Statistical differences between MRF-treated and MRF + DSF cotreated groups were indicated as follows: * *p* < 0.05; ** *p* < 0.01; *** *p* < 0.001 (MRF vs. MRF + DSF 12.5); # *p* < 0.05; ## *p* < 0.01; ### *p* < 0.001 (MRF vs. MRF + DSF 25); $$$ *p* < 0.001 (MRF vs. MRF + DSF 50).

**Figure 5:**
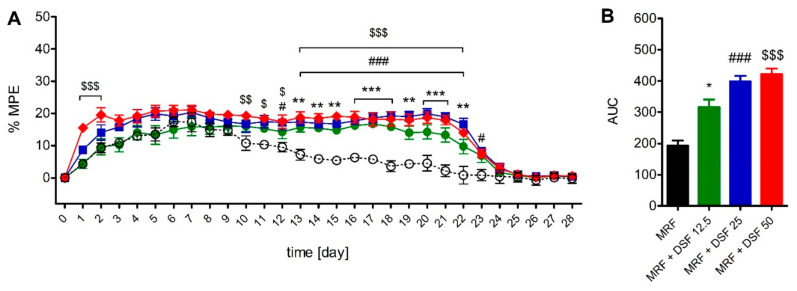
The time-course (**A**) and area under the curve (AUC) (**B**) of chronic disulfiram (DSF, 12.5-50 mg/kg, i.g.) on morphine-induced (MRF, 25 mg/kg, i.g) tolerance in the tail-flick test (*n* = 6). ○ MRF; ● MRF + DSF 12.5, ■ MRF + DSF 25, ♦ MRF + DSF 50. Control animals received either water for injection (MRF group control) or 0.1% methylcellulose (MRF + DSF group control). Baseline thresholds were measured at time 0. Drugs were delivered consecutively from day 1 until day 21. Drugs were discontinued on day 22 and nociceptive thresholds were measured until day 28. Results from the time-course curves were expressed as control-subtracted means ± SEM and analyzed with two-way ANOVA followed by Bonferroni’s post hoc test. Area under the curve (AUC) data were analyzed with one-way ANOVA. Statistical differences between MRF-treated and MRF + DSF cotreated groups were indicated as follows: * *p <* 0.05; ** *p* < 0.01; *** *p* < 0.001; (MRF vs. MRF + DSF 12.5); # *p* < 0.05; ### *p* < 0.001 (MRF vs. MRF + DSF 25); $ *p* < 0.05; $$ *p* < 0.01; $$$ *p* < 0.001 (MRF vs. MRF + DSF 50).

As indicated by two-way ANOVA, DSF altered nociceptive thresholds in MRF-treated rats when measured in the Randal–Selitto (F_3,580_ = 399.4; *p* < 0.001, Figure 4) and tail-flick (F_3,580_ = 137.1; *p* < 0.001, Figure 5) tests. The antinociceptive effect of MRF declined within 9 or 10 days of treatment (depending on the nociceptive test used), announcing the onset of tolerance. However, all DSF rescued the antinociceptive effect of MRF throughout the treatment regimen regardless of the dose used. In the Randal–Selitto test, rats receiving the lowest (12.5 mg/kg) and intermediate (25 mg/kg) doses of DSF, had higher mechanical thresholds from day 9 until day 22 of treatment (Figure 4A). Mechanical thresholds of rats receiving MRF with the highest dose of DSF (50 mg/kg) were higher within the first two days of treatment and from day 9 until day 23 when compared with MRF-treated rats. When the time-course area under curve (AUC) were calculated, mechanical nociceptive thresholds in MRF rats cotreated with 12.5, 25 and 50 mg/kg of DSF were higher by 2.7-, 3.5- and 6-fold, respectively, when compared to MRF alone (Figure 4B).

In the tail-flick test, rats treated with a combination of MRF and the lowest dose of DSF (12.5 mg/kg) showed elevated pain thresholds from day 13 until day 22 of treatment. Administration of 25 mg/kg DSF together with MRF resulted in a similar effect that surfaced from day 12 and lasted until day 23 (Figure 5A). Coadministration of the highest DSF dose tested (50 mg/kg) with MRF, resulted in a transient elevation of thermal thresholds in the first two days of cotreatment. Further enhancement of the antinociceptive effect of MRF was noted from day 10 post-treatment that persisted until day 22. Calculation of the AUC values yielded results showing that MRF groups cotreated with 12.5, 25 and 50 mg/kg of DSF, showed higher thermal pain thresholds by 0.63-, 1.06- and 1.18-fold than rats treated with MRF alone (Figure 5B).

### 2.5. The Effect of Disulfiram on µ-Opioid G-Protein Activation in Morphine-Treated Rats

**Figure 6:**
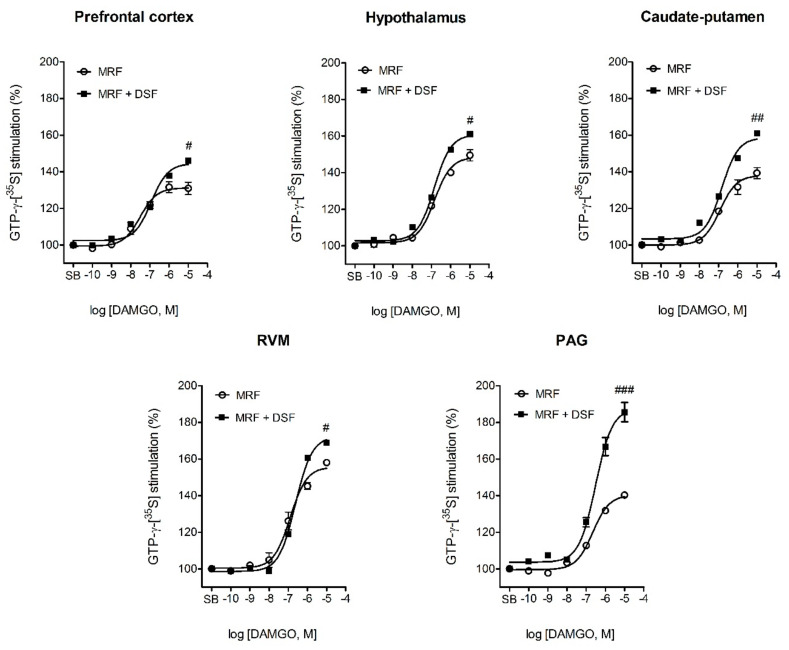
Concentration-response curves of DAMGO-stimulated G-protein activation in the prefrontal cortex, hypothalamus, caudate-putamen, rostral ventromedial medulla (RVM) and periaqueductal gray matter (PAG) of rats treated with morphine alone (MRF) or with morphine and disulfiram (MRF + DSF) after 1 day of treatment. Data were fitted with a one-site nonlinear regression model. Efficacy (E_max_) values were averaged from 6 rats (except for the RVM and PAG, where samples were pooled from 6 rats) and expressed as means ± SEM. Differences in E_max_ between MRF and MRF + DSF groups in each structure were evaluated with one-way ANOVA followed by Dunnett’s post hoc test. # *p* < 0.05; ## *p* < 0.01; ### *p* < 0.001 (MRF vs. MRF + DSF). SB-specific binding (no DAMGO added).

**Figure 7:**
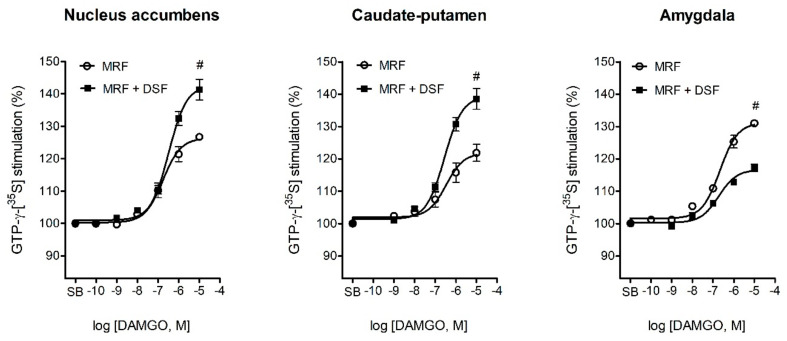
Concentration-response curves of DAMGO-stimulated G-protein activation in the nucleus accumbens, caudate-putamen and amygdala of rats treated with morphine alone (MRF) or with morphine and disulfiram (MRF + DSF) for 14 days. Data were fitted with a one-site nonlinear regression model. Efficacy (E_max_) values were averaged from 6 rats and expressed as means ± SEM. Differences in E_max_ between MRF and MRF + DSF groups in each structure were evaluated with one-way ANOVA followed by Dunnett’s post hoc test. # *p* < 0.05 (MRF vs. MRF + DSF). SB-specific binding (no DAMGO added).

Results showing DAMGO-stimulated µ-opioid receptor activation in different central nervous system (CNS) structures isolated from control, MRF and MRF + DSF-treated rats were listed in Table 2 and illustrated in Figure 6 and Figure 7. One-way ANOVA revealed a decrease in G-protein activation efficacy by DAMGO in CNS structures of rats treated with MRF as early as on the first day post-treatment (F_17,47_ = 25.9; *p* < 0.001) (Table 2, Figure 6). This effect was significant in the thalamus, nucleus accumbens, hypothalamus, caudate-putamen and periaqueductal gray matter (PAG). The most profound decreases were seen in the thalamus (180 ± 3.4 vs. 155 ± 2.2; *p* < 0.001), hypothalamus (177 ± 4.2 vs. 148 ± 2.2; *p* < 0.01) the PAG (162 ± 3.1 vs. 141 ± 1.5; *p* < 0.01) and nucleus accumbens (171 ± 3.6 vs. 155 ± 2.5; *p* < 0.01) as compared with control. A relatively modest decrease in G-protein activation was found in the caudate-putamen (147 ± 2.6 vs. 138 ± 2.6; *p* < 0.05). Similarly as in the MRF-treated rats, in the MRF + DSF group, a decrease in the efficacy of DAMGO was noted in the thalamus (180 ± 3.4 vs. 157 ± 2.2; *p* < 0.001), hypothalamus (177 ± 4.2 vs. 158 ± 2.24; *p* < 0.01) and nucleus accumbens (171 ± 3.6 vs. 153 ± 1.8; *p* < 0.01) (F_17,47_ = 23.9; *p* < 0.001). However, a decrease in µ-opioid receptor stimulation efficacy by DAMGO was slightly less pronounced in the hypothalamus of MRF + DSF rats than rats treated only with MRF (158 ± 2.24 vs. 148 ± 2.2; *p* < 0.05). More importantly, in some structures such as the prefrontal cortex, caudate-putamen, rostral ventromedial medulla (RVM) or PAG an 8.4% (*p* < 0.05), 13% (*p* < 0.01), 5.7% (*p* < 0.05) and 31.9% (*p* < 0.001) increase in µ-opioid receptor activation efficacy was detected as compared with MRF-treated rats. These values were also significantly greater than control animals.

At day 14 of treatment, a visible reduction in µ-opioid receptor activity relative to the control was noted in all analyzed structures for both the MRF (F_17,47_ = 21.2; *p* < 0.001) and MRF + DSF (F_17,47_ = 24.6; *p* < 0.001) groups (Table 2, Figure 7). However, DAMGO stimulated µ-opioid receptor activity more effectively in the nucleus accumbens (139 ± 2.6 vs. 120 ± 1.8; *p* < 0.05) and caudate-putamen (138 ± 1.9 vs. 120 ± 1.6; *p* < 0.05) of MRF + DSF rats when compared with rats chronically treated with MRF alone. Of note, in the MRF only group, higher µ-opioid receptor G-protein activation was detected in the amygdala than in the MRF + DSF group (130 ± 4.8 vs. 116 ± 1.5; *p* < 0.05).

## 3. Discussion

In the present study, we aimed to verify the hypothesis that disulfiram (DSF) enhances µ-opioid receptor G-protein signaling, which could in part contribute to the augmentation of morphine (MRF) antinociception and abolition of morphine tolerance development. A single bolus of DSF alone produced meager but significant antinociceptive action (Figure 1), which does not seem to be related to direct opioid receptor activation. As evidenced by our recent observations, although DSF alone produced potent enhancement of G-protein activity in the [^35^S]GTPγS assay, this action was not reversed by NTX (Figure S1A). Moreover, DSF altered neither potency nor efficacy of MRF-induced receptor stimulation in brain homogenates from naïve rats (Figure S1B). Thus, the pharmacological interplay between DSF and MRF could involve indirect mechanisms. The conducted behavioral assays confirmed that DSF rescued the antinociceptive effectiveness of chronically administered MRF (Figure 4 and Figure 5). This observation correlated with results from the functional ex vivo assay, where µ-opioid receptor signaling was not fully abolished following chronic MRF treatment (Table 2, Figure 7).

The first report describing the potential use of DSF as an adjunctive medication to pain management therapy was released in the late 1970s by Hosobuchi and Wemmer [18]. In that particular investigation, intractable pain patients experienced potentiation of PAG stimulation-induced analgesia (SIA) when concomitantly receiving DSF. Additionally, DSF provided long-standing protection against the development of analgesic tolerance. Moreover, DSF alone potently decreased opioid demand when SIA was discontinued [30]. These findings are in conformity with our observations as we also saw synergistic potentiation of MRF antinociception even after a single dose of DSF (Figure 2, Table 1). Moreover, the effect of both drugs was NTX-reversible, confirming the contribution of opioid receptor signaling (Figure 3).

The notion that µ-opioid receptors may play a vital role in the antinociceptive effect of DSF is derived from the well-acknowledged fact that they modulate the activity of pain-inhibitory neurons in the ventrolateral PAG (vlPAG). Namely, these neurons are under tonic inhibition from GABAergic interneurons, which is suppressed by µ-opioid receptor activation. This subsequently activates glutamatergic transmission in the RVM, leading to pain suppression [31,32]. Our investigation revealed a significant increase in µ-opioid receptor G-protein activation 23 h following drug delivery in the PAG of DSF and MRF cotreated rats compared with MRF alone. A similar trend was observed when rats receiving concomitant DSF and MRF treatment were compared with control animals. Similar results were also obtained for the RVM and prefrontal cortex, which both send their projections to the PAG [33] (Table 2, Figure 6). One might thus expect enhanced µ-opioid-dependent suppression of GABA release following DSF treatment and the ability of naltrexone to negate this effect when a mechanical stimulus is applied.

It seems that potentiation of MRF antinociception by acute DSF delivery was restricted to supraspinal sites, which are responsible for mechanical pain transmission. Morphine, apart from having a direct inhibitory effect on primary afferent neuron excitability, also increases output from the PAG to the RVM by decreasing GABAergic transmission. As confirmed recently, mechanical pain is modulated by presynaptic enkephalin and GABA release from dorsal horn interneurons located mainly in laminae II and III. Their suppression increases mechanical but not thermal sensitivity due to a limited number of heat-sensitive C-fibers receiving input from enkephalinergic interneurons in lamina I [34,35]. Thus, it is possible that DSF enhances enkephalin release from dorsal horn interneurons, causing presynaptic inhibition of mechanosensory neurons. Especially, when DSF was previously shown to increase plasma Met-enkephalin levels in dogs subjected to an acute ethanol challenge [36].

As mentioned earlier, our study has shown major changes in µ-opioid receptor G-protein activation as early as on day 1 of MRF treatment (Table 2, Figure 6). Structures that were mostly affected included the thalamus, hypothalamus, PAG, nucleus accumbens and to a lesser extent the caudate-putamen. The first three structures are involved in nociceptive processing and their stimulation has long been acknowledged to trigger pain-relief in an opioid-related manner [37,38,39]. Moreover, as we have shown earlier, hypothalamic, thalamic and PAG µ-opioid receptor G-protein activity is decreased in a mouse line refractory to opioid-induced analgesia [40]. Of note, the early enhancement of G-protein activity in these structures does not contribute significantly to the long-lasting effect of DSF, as the differences in G-protein activity between MRF-treated and DSF and MRF cotreated rats diminished at day 14. A noticeable decline in DAMGO-stimulated G_i/0_-protein activation upon chronic MRF exposure was evidenced before in many of the brain structures studied. This implicates biased functional signaling as the mechanism responsible for the appearance of MRF tolerance [41,42].

As evidenced before, chronic MRF treatment induces μ-opioid receptor adaptive modifications responsible for the decline in drug efficacy over time. It has been reported that chronic MRF treatment induces tolerance that is at least in part owed to desensitization effected by weak receptor internalization and recycling as well as serine 375 phosphorylation [43,44,45]. The phenomenon of tolerance is also thought to be directly linked to regionally specific G_i/0_ uncoupling, where a progressive reduction in signal transduction is observed without major changes in receptor density [42,46,47]. Stimulation of cAMP production (or cAMP overshoot) by MRF speaks in favor of G_i/0_ uncoupling and preferential coupling of the receptor to the Gs stimulatory subunit [48,49]. Additionally, a 10-day chronic MRF exposure was shown to increase the G_s_/G_i/0_ protein ratio in forebrain cell membrane fractions, which correlated with a trend toward lower DAMGO efficacy [50]. This observation is in line with our behavioral data, where MRF efficacy was compromised at this timepoint.

Recently, the role of the striatal nuclei comprising the nigrostriatal dopamine pathway were indicated in nociceptive processing and pain perception owing to their high density of opioid receptors [51,52,53]. Similarly as in the PAG, chronic DSF exposure was evidenced to attenuate GABA release and decrease glutamate uptake in the striatum [27,54]. Our study provided further functional evidence that upregulation of µ-opioid receptor activity by DSF in the nucleus accumbens and caudate-putamen may correlate with sustained MRF efficacy. Especially, it was shown that µ-opioid receptor-mediated G-protein activation in the striatum is more resistant to desensitization than in the brainstem nuclei [55,56]. Observations that some forms of antinociception rely on nucleus accumbens opioid receptors only when µ-opioid receptors have not yet undergone downregulation further supports their importance of the striatum in chronic opioid efficacy [57].

As the most meaningful changes in µ-opioid receptor signaling were detected in structures of the dopamine circuit, the role of this monoamine in the behavioral effects of DSF cannot be ruled out. Especially when DSF and its active metabolites inhibit β-hydroxylase activity, elevated dopamine levels suppress GABA_A_ receptor signaling in the PAG and trigger upregulation of µ-opioid receptor mRNA in the nucleus accumbens [27,54,58,59,60,61]. It is possible that in our behavioral investigation, NTX executes its pronociceptive effects by reducing DSF-induced dopamine release. This effect was previously observed in the nucleus accumbens in male rats either exposed to a pleasurable stimulus or challenged with amphetamine [62,63]. However, further studies are needed to confirm the role of dopamine signaling in the antinociceptive effect of DSF. Additionally, other clinically important implications such as increased likelihood of addiction to stimulants or the risk of drug-induced psychosis in schizophrenic patients need to be addressed as well [20,64].

Interestingly, the amygdala of chronic MRF-treated rats was the only structure where an increase in μ-opioid receptor signaling was detected. It is possible that sustained μ-opioid receptor activation in this brain region perpetuates the development of opioid dependence, as shown before [65,66]. As described in our earlier behavioral study [26], disulfiram abrogated MRF-induced dependence and withdrawal. The mechanistic underpinnings of this phenomenon are not yet known; however, the current study has shown that disulfiram pretreatment causes downregulation of μ-opioid receptor signaling in the amygdala (Table 2, Figure 7). Of course, other mechanisms may also contribute to the reversal of MRF preference and dependence. Interestingly, activation of amygdala neurokinin-1 (NK1) receptors was linked to MRF craving [67] and disulfiram was previously reported to reduce substance-P (SP) release [68].

It may seem that the side-effect profile of DSF, including hepatic, psychiatric and neurotoxic complications [69], may potentially render this drug unsuitable for long-term support of opioid-based pain management—especially when the most recent investigation points to the possibility of liver damage in MRF-treated, alcohol-naïve animals [70]. Firstly, however, most of the adverse reactions were seen in patients with alcohol use disorder. Secondly, the most recent study employed a high-dose DSF treatment scheme, as only the 100 mg/kg dose produced meaningful extension of MRF antinociception when delivered intraperitoneally. Such high doses of DSF carry a greater risk of hepatotoxicity and may additionally cause disturbances in catecholamine levels, leading to neurotoxic effects [21], whereas our study demonstrated that 25 mg/kg DSF is sufficient enough to exert an effect of similar magnitude when given orally, which potentially lowers the risk of hepatitis and/or neurotoxicity. Thus, low-dose oral DSF may still be considered a valuable asset to MRF-based pain relief strategy owing to its robust and long-lasting suppression of analgesic tolerance and lower risk of side-effects.

## 4. Materials and Methods

### 4.1. Animals and Husbandry

The study was conducted in compliance with the guidelines published in the European directive 2010/63/EU on the protection of animals used for scientific purposes. The protocol was approved by the Ethical Committee for Experiments on Small Animals, Medical University of Warsaw (permit numbers: 64/2013 and 101/2016). Outbred, male Cmdb:Wi rats (270–320 g) were housed in a room maintained at a temperature of 20 ± 2 °C, under 12–12 h light–dark cycle. Experimental groups consisted of 6 rats. Animals had free access to food and water.

### 4.2. Chemicals and Supplies

Disulfiram (DSF), morphine sulfate and water for injection were purchased from Polfa (Warsaw, Poland). Guanosine 5′-[γ-thio]triphosphate (GTPγS), guanosine 5′-[γ-thio]triphosphate tetralithium salt (GTP), Guanosine 5′-diphosphate sodium salt (GDP), ethylenoglycol-bis(f6-aminoethyl-ether)-N,N,N′,N′-tetraacetic acid (EGTA), ethylenediaminetetraacetic acid (EDTA), DAla^2^,NMePhe^4^,Gly^5^-ol enkephalin (DAMGO), naloxone hydrochloride, cOmplete™, Mini EDTA-free Protease Inhibitor Cocktail tablets and methylcellulose were purchased from Sigma Aldrich (Munich, Germany). [^35^S]GTPγS (1250 Ci/mmol), UniFilter-96 GF/B Barex Microplates, OptiPhase Supermix Cocktail scintillant were purchased from Perkin Elmer (Waltham, MA, USA). K_2_EDTA-coated lavender tubes were obtained from Sarstedt (Numbrecht, Germany). Tubes containing zirconium 1.4 mm beads were obtained from DNA Gdansk, Poland. MgCl_2_, NaCl were purchased from Avantor Performance Materials Poland S.A. (Gliwice, Poland).

### 4.3. Drug Administration

All drugs were administered by oral gavage in a volume of 1 mL/kg. Morphine (MRF, 25 mg/kg), naltrexone (NTX, 25 mg/kg) were dissolved in water for injection. Disulfiram (DSF, 12.5, 25 and 50 mg/kg) was suspended in 0.1% methylcellulose solution as described previously [26]. In experiments where DSF was delivered as a single bolus, control animals received a 0.1% methylcellulose solution. In acute cotreatment experiments, control animals received water for injection (MRF and MRF + NTX group control) or 0.1% methylcellulose (control for groups treated and cotreated with DSF). DSF or NTX were administered 10 min before MRF. In the NTX + DSF + MRF group, NTX was delivered 10 min before DSF and MRF was administered 10 min following DSF. The following experimental groups were included in the chronic behavioral assays: MRF (water + 25 mg/kg morphine), MRF + DSF 12.5 (25 mg/kg morphine + 12.5 mg/kg disulfiram), MRF + DSF 25 (25 mg/kg morphine + 25 mg/kg disulfiram), MRF + DSF 50 (25 mg/kg morphine + 50 mg/kg disulfiram). Control animals received 0.1% methylcellulose (MRF + DSF group control) or water for injection (MRF group control). In behavioral experiments, all drugs were administered for 21 consecutive days. All drugs were discontinued on day 22 and nociceptive thresholds were measured until day 28. In experiments involving tissue harvest for the functional [^35^S]GTPγS assay, drugs or vehicle (0.1% methylcellulose) were administered either for 1 or 14 days and animals were sacrificed 23 h later.

### 4.4. Behavioral Testing

Nociceptive thresholds were measured in the Randal–Selitto and tail-flick tests as described previously [71]. In the single bolus experiments mechanical a thermal nociceptive thresholds were measured before drug/vehicle administration and every 30 min for 3.5 h. A 1 min interval was ensured between both types of tests. Animals were returned back to their home cages between testing at each timepoint. In chronic drug delivery experiments, single measurements were taken in individual animals every day, starting at 10.00 a.m. at 23 h postadministration. After discontinuation of drug treatment at day 21, behavioral testing continued until day 28. The tail-flick test was performed first. Changes in pain thresholds for each animal were calculated as % maximum possible effect (%MPE) using the following formula (Formula (1)):

%MPE = [T_2_ − T_1_]/[C_off_ − T_1_] × 100% (1)

where T_1_—baseline latency before treatment; T_2_—post-treatment latency; C_off_—cut-off time.

### 4.5. Measurement of Thermal Thresholds

Thermal thresholds were measured in the tail-flick test according to the thermal stimulation of the tail method by D’Amour and Smith [72] using the I.R. Tail-Flick Unit (Ugo Basile, Comerio, Italy). Briefly, rats were gently restrained by hand. Then, radiant heat was directed onto the ventral aspect of the tail, 4–5 cm from the tip. A positive nocifensive reaction was a vigorous removal of the tail from the heat source, which automatically terminated the test and recorded tail-flick latency (in seconds). Light intensity was set to elicit a baseline response of ca. 6 s. Cut-off time was set to 20 s to avoid burns to the tail.

### 4.6. Measurement of Mechanical Thresholds

Mechanical thresholds were determined in the Randal–Selitto paw pressure test originally described previously [73] with some modifications. Briefly, rats were restrained by hand and increasing pressure was applied to the dorsal surface of the hindpaw at a rate of 32 g/s. Nociceptive thresholds were defined as the force (in grams) that evoked an attempt to withdraw the paw. The cut-off pressure was set to 480 g. 

### 4.7. Homogenate Preparation

Rats from the MRF and MRF + DSF 25 groups from day 1 and day 14 of treatment were sacrificed by decapitation under deep isoflurane anesthesia. Their brains were carefully removed and selected CNS structures (frontal cortex, prefrontal cortex, thalamus, hypothalamus, nucleus accumbens, caudate-putamen, rostral ventromedial medulla (RVM), periaqueductal gray matter (PAG) and amygdala) were dissected on ice using the Alto Stainless Steel Coronal 0.5 mm Brain Matrix. The tissue was immediately frozen on dry ice following dissection and stored at −80 °C until use. After thawing, tissues were homogenized with the FastPrep-24™ Classic Instrument disintegrator (MP Biomedicals, Santa Ana, CA, USA) for 20 s at 4.0 m/s in 30 vol./wet weight of homogenization buffer (50 mM Tris-HCl, 1 mM EDTA and 1× cOmplete Mini, EDTA-free Protease Inhibitor Cocktail, pH = 7.4) in zirconium bead tubes (bead diameter: 1.4 mm in diameter) (DNA Gdansk, Poland). Periaqueductal gray matter and RVM regions isolated from 6 rats were pooled. Next, homogenates were centrifuged for 30 min at 22,000× *g* in 4 °C. The resulting pellet was suspended in 30 vol. of 50 mM Tris-HCl, pH = 7.4 and incubated for 30 min at room temperature to dissociate endogenous ligands. The centrifugation step was repeated and the final pellet was suspended in 10 vol. of 50 mM Tris-HCl, pH = 7.4. Tissues were stored at −80 °C until use. Protein concentration was assessed with the Micro BCA Protein Assay Kit (Thermo Fisher Scientific, Waltham, MA, USA).

### 4.8. [35. S]GTPγS Binding Assay

The assay was carried out as previously described [40]. Homogenates (15μg/mL) were incubated in triplicate with 0.8 nM [^35^S]GTPγS and stimulating ligand (10^−10^ M–10^−5^ M) in 50 mM Tris-HCl, pH = 7.4 binding buffer supplemented with 1 mM EGTA, 3 mM MgCl_2_, 100 mM NaCl, 30 µM GDP for 90 min at 30 °C. To evaluate opioid-receptor related changes in G-protein activation in chronic DSF and MRF cotreatment experiments, DAMGO (10^−10^ M–10^−5^ M) was used as the stimulating ligand. Total assay volume was 250 µL. Unlabeled GTPγS was used to determine nonspecific binding. Samples were rapidly vacuum filtered through 96-well Unifilter Plates presoaked with 50 mM Tris-HCl, pH = 7.4 (Perkin Elmer, Waltham, MA, USA) using the FilterMate Harvester (Perkin Elmer, Waltham, MA, USA). Every filter-coated well was then washed with 2 mL of wash buffer (50 mM Tris-HCl, pH = 7.4) to separate bound from free radioligand. After drying overnight at room temperature, 45µl of OptiPhase Supermix Cocktail scintillant (Perkin Elmer, Waltham, MA, USA) was added to each filter well and left for 6 h to equilibrate. Filter-bound radioactivity was counted in a Trilux MicroBeta^2^ counter (Perkin Elmer, Waltham, MA, USA). Curves were fitted with a one-site non-linear regression model available from GraphPad Prism, version 5.03 software (GraphPad Software, San Diego, CA, USA, https://www.graphpad.com/ (accessed on 20 June 2019)). Efficacy (E_max_) and potency (EC_50_) of DAMGO stimulation in various brain structures were calculated and expressed as means ± SEM from 6 animals.

**Figure 8:**
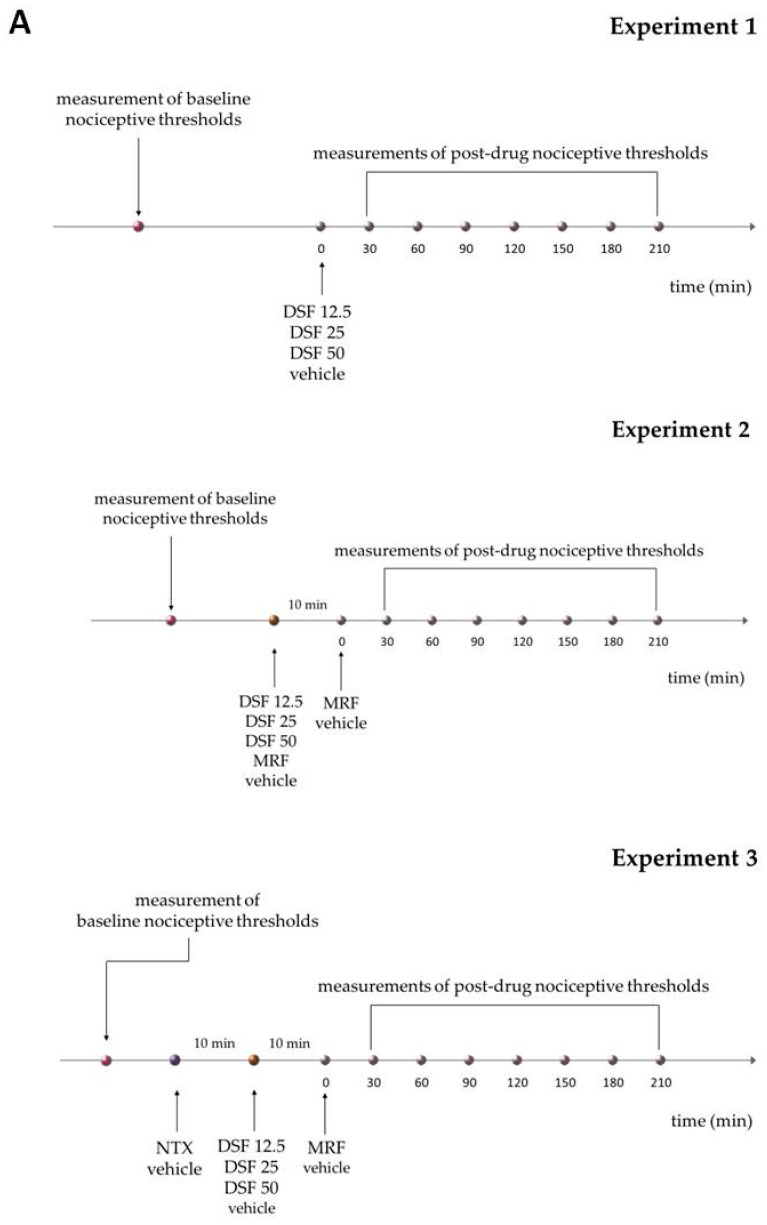

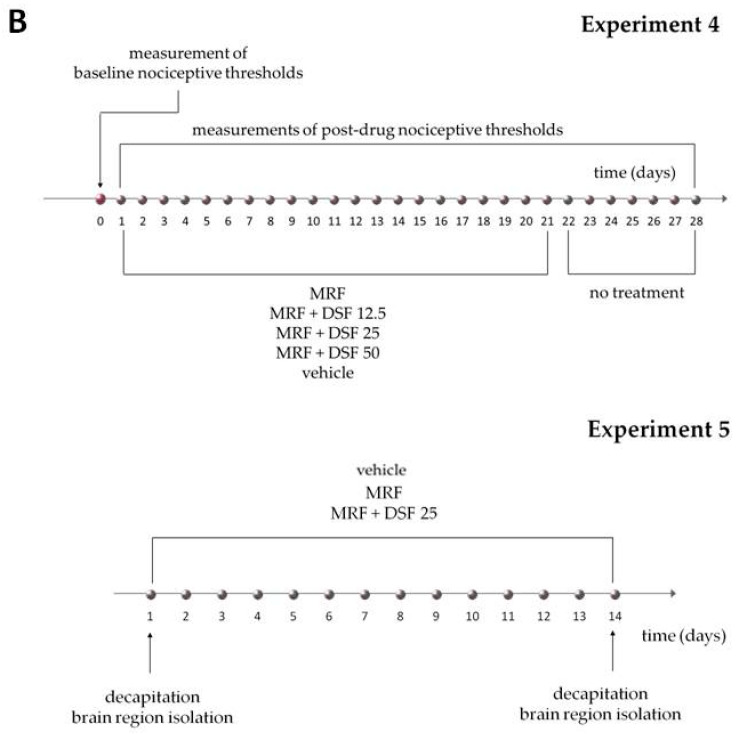
Scheme of the study design. Panel (**A**,**B**) represent acute and chronic experiments, respectively.

A scheme representing the study design is included below (Figure 8).

### 4.9. Statistical Analysis

Behavioral data from chronic cotreatment experiments along with data involving NTX-reversal of acute MRF and DSF effects, were reported as mean control-subtracted values ± SEM. Other acute treatment data were shown along with respective controls and expressed as mean ± SEM. Results from time-course curve experiments were analyzed with two-way ANOVA, followed by Bonferroni’s post hoc test. Area under the time-course curve (AUC) data were analyzed with either the nonparametric Kruskal–Wallis test or one-way ANOVA followed by Dunnet’s post hoc test. The nature of MRF + DSF combination index (CI) was calculated at peak effect corresponding to 60 min postadministration according to the Bliss Independence model [74] (2):

CI = (E_A_ + E_B_ − E_A_ × E_B_)/E_AB_ (2)

where E_A_—fractional effect of drug A; E_B_—fractional effect of drug B; E_AB_—fractional effect of drug combination. A CI < 1 indicates synergy, CI = 1 an additive effect and CI > 1 antagonism.

Binding data were fitted with a one-site nonlinear regression model provided by GraphPad Prism, version 5.03 software for Windows (GraphPad Software, San Diego, CA, USA, www.graphpad.com) (accessed on 20 June 2019). Efficacy (E_max_) and potency (EC_50_) were calculated using Equation (3):

Y = Bottom + (T − B)/(1 + 10^logEC50−X^) (3)

where Y—% stimulation, B—% stimulation at bottom plateau, T—% stimulation at top plateau, X—ligand concentration in log units, logEC_50_—ligand concentration at half-maximal stimulation.

E_max_ and pEC_50_ values were expressed as means ± SEM from averaged data collected from 6 rats. Differences in potency and efficacy of DAMGO in different CNS structures in MRF + DSF, MRF and control rats at day 1 and 14 were evaluated with one-way ANOVA. As G-protein stimulation efficacy was comparable in both MRF and MRF + DSF controls (water for injection and 0.1% methylcellulose), the results were combined.

## 5. Conclusions

Our study is the first to describe the possible mechanism contributing to the maintenance of morphine antinociception by low-dose DSF. We proposed the importance of the partial rescue of supraspinal µ-opioid G-protein signaling by DSF in the striatum. Of note, the mechanism presented in the current study constitutes only a small piece of the puzzle and other yet unknown mechanisms are certainly involved. It should be emphasized that DSF affects a plethora of physiological pathways. Nevertheless, the presented research is a first but significant step towards considering low-dose DSF as a possible adjunct agent for opioid pain management.

## Figures and Tables

**Table 1:** Combination index values (CI ^1^) for morphine (MRF, 25 mg/kg, i.g.) and disulfiram (DSF, 12.5–50 mg/kg, i.g.) cotreatment.

Drug Combination	Randal–Selitto Test	Tail-Flick Test
MRF + DSF 12.5	0.69	1.12
MRF + DSF 25	0.61	1.04
MRF + DSF 50	0.66	0.9

^1^ CI < 1–synergism; CI = 1–addition; CI > 1–antagonism.

**Table 2:** DAMGO-stimulated G-protein activation in brain structures of MRF-treated and MRF + DSF cotreated rats at days 1 and 14.

	E_max_ ± SEM (%)				
CNS Structure	Control	DAY 1		DAY 14	
		DSF + MRF	MRF	DSF + MRF	MRF
frontal cortex	140 ± 2.9	140 ± 2.4	134 ± 3.4	121 ± 2.9 **	129 ± 2.1*
thalamus	180 ± 3.4	157 ± 2.2 ***	155 ± 2.2 ***	136 ± 1.9 ***	139 ± 3.0 ***
hippocampus	139 ± 2.8	131 ± 2.6	134 ± 2.9	120 ± 1.2 **	120 ± 2.4 **
prefrontal cortex	133 ± 3.0	142 ± 1.9 * #	131 ± 2.5	122 ± 2.3 *	118 ± 2.6 *
nucleus accumbens	171 ± 3.6	153 ± 1.8 **	155 ± 2.5 *	139 ± 2.6 #	120 ± 1.8 ***
hypothalamus	177 ± 4.2	158 ± 2.24 ** #	148 ± 2.2 **	113 ± 1.0 ***	116 ± 2.0 ***
caudate-putamen	147 ± 2.6	156 ± 2.3 * ##	138 ± 2.6 *	138 ± 1.9 #	120 ± 1.6 ***
RVM	156 ± 2.0	168 ± 1.8 ** #	159 ± 2.3	134 ± 2.5 **	132 ± 2.3 ***
amygdala	151 ± 3.4	145 ± 2.9	141 ± 2.4	116 ± 1.5 ***	130 ± 4.8 * #
PAG	162 ± 3.1	186 ± 5.5 ** ###	141 ± 1.5 **	133 ± 3.3 ***	136 ± 3.5 **

Rats were treated with MRF (25 mg/kg, i.g.) or MRF + DSF (25 mg/kg, i.g.). Data were fitted with a one-site nonlinear regression model and efficacy (E_max_) was calculated and expressed as means ± SEM from averaged data collected from 6 rats. Differences in E_max_ of DAMGO in different CNS structures in MRF + DSF, MRF and control (0.1% methylcellulose) rats at day 1 and 14, were evaluated with one-way ANOVA followed by Bonferroni’s post hoc test. Statistical significance was expressed as follows: * *p* < 0.05; ** *p* < 0.01; *** *p* < 0.001 vs. control; # *p* < 0.05; ## *p* < 0.01; ### *p* < 0.001 (DSF + MRF vs. MRF). RVM-rostral ventromedial medulla; PAG-periaqueductal gray matter.

## Data Availability

Data is contained within the article or supplementary material.

## Supplementary Materials

The following are available online at https://www.mdpi.com/article/10.3390/ijms22084057/s1.
