# Supplementary material for: Disulfiram Abrogates Morphine Tolerance—A Possible Role of µ-Opioid Receptor-Related G-Protein Activation in the Striatum
Source: Int J Mol Sci. 2021 Apr 14;22(8):4057. doi: 10.3390/ijms22084057 (PMC8071001; doi:10.3390/ijms22084057)
Supplement: Supplementary file 1 [file ijms-22-04057-s001.pdf]

Supplementary Data

The stimulating effect of DSF in the [^{35}S]GTPγS binding assay

To assess the possible stimulating effect of disulfiram (DSF) on G-protein activity, DSF (10^{-10} – 10^{-5}M) was incubated for 90min. at 30°C with brain homogenates (15μg/ml) from naïve rats in a 50mM Tris-HCl, pH = 7.4 binding buffer, containing 1mM EGTA, 3mM $MgCl_2$, 100mM NaCl and 30μM GDP. To investigate the putative involvement of the opioid system in DSF-stimulated G-protein activation, naltrexone (10^{-6}M, NTX) was added to the incubation mixture. To evaluate the possible modulating effect of DSF on MRF-induced opioid receptor stimulation, MRF (10^{-10} – 10^{-5}M) was incubated with an EC_{80} background DSF concentration (5.7 x 10^{-7}M). The effect of MRF on DSF-induced stimulation was assessed by incubating DSF (10^{-10} – 10^{-5}M) with an EC_{80} background MRF concentration (10^{-7}M). Unlabeled GTPγS was used to determine non-specific binding. The reaction mixture (250μl) was rapidly vacuum filtered through 96-well Unifilter Plates presoaked with 50mM Tris-HCl, pH = 7.4 (Perkin Elmer, USA) with the FilterMate Harvester (Perkin Elmer, USA). Every filter-coated well was then washed with 2 ml of wash buffer (50 mM Tris-HCl, pH = 7.4) to separate bound from free radioligand. After drying overnight at room temperature, 45μl of OptiPhase Supermix Cocktail scintillant (Perkin Elmer, USA) was added to each filter well and left for 6h to equilibrate. Filter-bound radioactivity was counted in a Trilux MicroBeta^2 counter (Perkin Elmer, USA). Curves were fitted with a one-site non-linear regression model available from GraphPad Prism 5.0 software (GraphPad Software, San Diego California USA, www.graphpad.com). Efficacy (E_{max}) and potency (pEC_{50}) values from 3 separate experiments were averaged, expressed as means ± SEM and compared with one-way ANOVA.

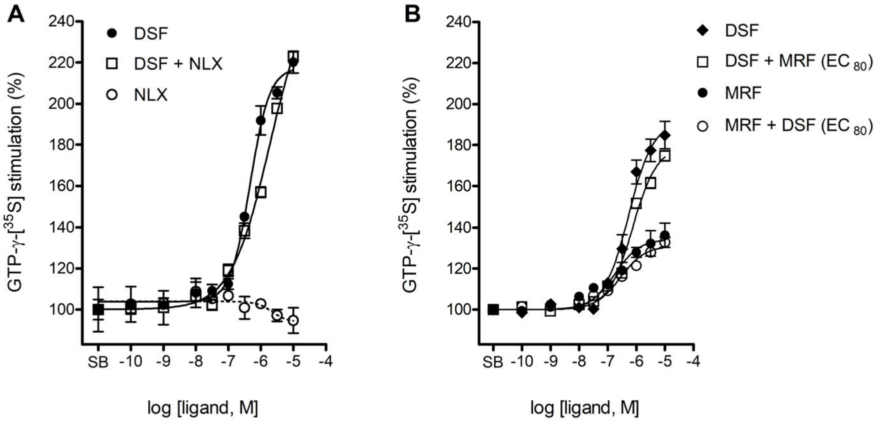

Fig. S1 (A) The stimulating effect of disulfiram (DSF, 10^{-10} – 10^{-5}M) and the effect of naltrexone (NTX, 10^{-6}M) on disulfiram (DSF)-stimulated G-protein activity in brain membrane homogenates from naïve rats. (B) The effect of morphine (MRF, 10^{-7}M) on DSF (10^{-10} – 10^{-5}M) and the effect of DSF (5.7 x 10^{-7}M) on MRF-stimulated G-protein activation in homogenates from naïve rats. Results are expressed as means ± SEM from 3 independent experiments. Efficacy (E_{max}) and potency (pEC_{50}) were calculated from the non-linear sigmoidal dose-response model and analyzed with one-way ANOVA. SB – specific binding (no ligand added).

DSF stimulates G-protein activity in an opioid-independent manner, but does not alter morphine stimulation

DSF induced [^{35}S]GTPγS stimulation with high efficacy (218 ± 8.7%) and submicromolar potency (pEC_{50} = 6.3 ± 0.05). NLX did not affect the efficacy ($F_{1,54}$= 0.42; $p > 0.05$) or potency ($F_{1,54}$ = 1.01; $p > 0.05$) of DSF-induced [^{35}S]GTPγS stimulation (Fig. S1). Additionally, DSF did not affect the efficacy ($F_{1,86}$ = 1.1; $p > 0.05$) or potency ($F_{1,86}$ = 0.95; $p > 0.05$) of MRF-induced G-protein stimulation. Subsequently, MRF did not affect the DSF-stimulated G protein activation in terms of efficacy ($F_{1,76}$ = 3.1; $p > 0.05$) or potency ($F_{1,76}$ = 1.72; $p > 0.05$).
